# Supplementary material for: Feasibility, acceptability and preliminary effectiveness of a community-based group psychosocial support model for conflict survivors in Colombia: An assessment of in-person and remote intervention modalities during the COVID-19 pandemic
Source: Glob Ment Health (Camb). 2024 Apr 30;11:e61. doi: 10.1017/gmh.2024.50 (PMC11106545; doi:10.1017/gmh.2024.50)
Supplement: James et al. supplementary material [file S2054425124000505sup001.docx]

**Supplementary Material 1.** ITT and PP pre and post intervention means and t-tests for primary outcomes in the in-person and remote trials.

|  |  | **ITT** | | | **PP** | | |
| --- | --- | --- | --- | --- | --- | --- | --- |
| *In-Person* | | *Pre* | *Post* | Pre-post t-test *p* value | *Pre* | *Post* | Pre-post t-test *p* value |
|  |  | Mean (SD) | Mean (SD) |  | Mean (SD) | Mean (SD) |  |
| Depression | Intervention | **1.18 (0.60)** | **0.94 (0.63)** | **0.02** | **1.19 (0.60)** | **0.88 (0.61)** | **0.01** |
|  | Control | 0.98 (0.55) | 0.90 (0.57) | 0.36 | 0.98 (0.55) | 0.90 (0.57) | 0.36 |
| Anxiety | Intervention | **1.02 (0.71)** | **0.76 (0.67)** | **0.02** | **0.95 (0.68)** | **0.65 (0.64)** | **0.02** |
|  | Control | 0.83 (0.62) | 0.78 (0.62) | 0.56 | 0.83 (0.62) | 0.78 (0.62) | 0.56 |
| PTSD | Intervention | **1.15 (0.64)** | **0.88 (0.59)** | **0.01** | **1.11 (0.57)** | **0.84 (0.54)** | **0.01** |
|  | Control | 1.05 (0.67) | 1.04 (0.62) | 0.88 | 1.05 (0.67) | 1.04 (0.62) | 0.88 |
| Generalized Distress | Intervention | **2.65 (0.85)** | **2.27 (0.77)** | **0.00** | **2.68 (0.81)** | **2.18 (0.75)** | **0.00** |
|  | Control | **2.62 (0.86)** | **2.32 (0.81)** | **0.02** | **2.62 (0.87)** | **2.32 (0.82)** | **0.02** |
| Functional Impairment | Intervention | 1.55 (0.46) | 1.52 (0.53) | 0.68 | 1.51 (0.43) | 1.43 (0.43) | 0.35 |
|  | Control | 1.48 (0.42) | 1.49 (0.42) | 0.81 | 1.48 (0.42) | 1.49 (0.42) | 0.81 |
| Community Resilience | Intervention | 3.44 (0.45) | 3.53 (0.39) | 0.17 | 3.49 (0.38) | 3.55 (0.38) | 0.42 |
|  | Control | 3.49 (0.39) | 3.54 (0.39) | 0.44 | 3.49 (0.39) | 3.54 (0.39) | 0.44 |
|  |  | **ITT** | |  | **PP** | |  |
| *Remote* | | *Pre* | *Post* | Pre-post t-test *p* value | *Pre* | *Post* | Pre-post t-test *p* value |
|  |  | Mean (SD) | Mean (SD) |  | Mean (SD) | Mean (SD) |  |
| Depression | Intervention | **1.12 (0.63)** | **0.82 (0.61)** | **0.02** | **1.17 (0.67)** | **0.80 (0.60)** | **0.02** |
|  | Control | **1.17 (0.59)** | **0.77 (0.64)** | **0.00** | **1.17 (0.59)** | **0.77 (0.64)** | **0.00** |
| Anxiety | Intervention | **0.96 (0.73)** | **0.65 (0.58)** | **0.02** | **1.01 (0.77)** | **0.62 (0.55)** | **0.02** |
|  | Control | **0.99 (0.67)** | **0.63 (0.64)** | **0.01** | **0.99 (0.67)** | **0.63 (0.64)** | **0.01** |
| PTSD | Intervention | **1.07 (0.71)** | **0.74 (0.69)** | **0.02** | **1.09 (0.70)** | **0.78 (0.73)** | **0.08** |
|  | Control | **1.07 (0.67)** | **0.60 (0.65)** | **0.00** | **1.07 (0.67)** | **0.60 (0.65)** | **0.00** |
| Generalized Distress | Intervention | **2.77 (0.74)** | **2.26 (0.60)** | **0.00** | **2.84 (0.79)** | **2.30 (0.65)** | **0.00** |
|  | Control | **2.67 (0.81)** | **2.23 (0.81)** | **0.01** | **2.67 (0.81)** | **2.23 (0.81)** | **0.01** |
| Functional Impairment | Intervention | 1.68 (0.65) | 1.58 (0.59) | 0.40 | 1.82 (0.71) | 1.64 (0.60) | 0.27 |
|  | Control | 1.65 (0.59) | 1.58 (0.73) | 0.62 | 1.65 (0.59) | 1.58 (0.73) | 0.62 |
| Community Resilience | Intervention | 3.42 (0.55) | 3.59 (0.57) | 0.15 | 3.34 (0.64) | 3.58 (0.66) | 0.16 |
|  | Control | 3.50 (0.42) | 3.43 (0.66) | 0.59 | 3.50 (0.42) | 3.43 (0.66) | 0.59 |

 Note: Depression, anxiety, and PTSD measured using Likert scales from 0-3; Generalized distress 1-5; Wellbeing 1-10; Functional impairment 1-5; Community resilience 1-4.

**Supplementary Material 2.** We performed a sensitivity analysis for different PP thresholds (attending at least 0 sessions to at least 6 sessions in total) to identify if the change in PP analysis approach resulted in relevant changes to our analysis and results. Consistent statistically significant results were found for depression, anxiety, and PTSD outcomes across all attendance cut-offs in the in-person modality, with small to moderate effect sizes (ɳ^2^_p depression_ = 0.04 to 0.10; ɳ^2^_p anxiety_ = 0.03 to 0.06; ɳ^2^_p PTSD_ = 0.04 to 0.07). There were no consistent significant effects in the sensitivity analysis for the remote modality.

| **In-Person Remote Results** | | | |
| --- | --- | --- | --- |
| **Minimum Sessions Attended** | **Treatment Effect** | **P value** | **Partial Eta Squared** |
| Depression |  |  |  |
| ITT Analysis | **-0.18** | **0.03** | **0.03** |
| 1 + Session | **-0.24** | **0.01** | **0.05** |
| 2 + Sessions | **-0.24** | **0.01** | **0.05** |
| 3 + Sessions | **-0.21** | **0.02** | **0.04** |
| 4 + Sessions | **-0.25** | **0.01** | **0.06** |
| 5 + Sessions | **-0.31** | **0.00** | **0.09** |
| 6 + Sessions | **-0.27** | **0.01** | **0.06** |
| Anxiety |  |  |  |
| ITT Analysis | **-0.19** | **0.03** | **0.03** |
| 1 + Session | **-0.27** | **0.01** | **0.05** |
| 2 + Sessions | **-0.26** | **0.01** | **0.05** |
| 3 + Sessions | **-0.22** | **0.02** | **0.04** |
| 4 + Sessions | **-0.26** | **0.01** | **0.06** |
| 5 + Sessions | **-0.24** | **0.02** | **0.05** |
| 6 + Sessions | **-0.23** | **0.05** | **0.04** |
| PTSD |  |  |  |
| ITT Analysis | **-0.27** | **0.00** | **0.05** |
| 1 + Session | **-0.29** | **0.00** | **0.06** |
| 2 + Sessions | **-0.29** | **0.01** | **0.06** |
| 3 + Sessions | **-0.27** | **0.01** | **0.05** |
| 4 + Sessions | **-0.29** | **0.01** | **0.06** |
| 5 + Sessions | **-0.36** | **0.00** | **0.09** |
| 6 + Sessions | **-0.37** | **0.00** | **0.08** |
| **Remote Modality Results** | | | |
| **Minimum Sessions Attended** | **Treatment Effect** | **P value** | **Partial Eta Squared** |
| Depression |  |  |  |
| ITT Analysis | 0.09 | 0.50 | 0.00 |
| 1 + Session | 0.05 | 0.70 | 0.00 |
| 2 + Sessions | 0.01 | 0.92 | 0.00 |
| 3 + Sessions | -0.02 | 0.90 | 0.00 |
| 4 + Sessions | 0.04 | 0.81 | 0.00 |
| 5 + Sessions | 0.03 | 0.85 | 0.00 |
| 6 + Sessions | 0.06 | 0.69 | 0.00 |
| Anxiety |  |  |  |
| ITT Analysis | 0.03 | 0.81 | 0.00 |
| 1 + Session | -0.01 | 0.97 | 0.00 |
| 2 + Sessions | -0.03 | 0.84 | 0.00 |
| 3 + Sessions | -0.08 | 0.61 | 0.00 |
| 4 + Sessions | -0.04 | 0.79 | 0.00 |
| 5 + Sessions | -0.06 | 0.72 | 0.00 |
| 6 + Sessions | -0.12 | 0.46 | 0.01 |
| PTSD |  |  |  |
| ITT Analysis | 0.16 | 0.22 | 0.02 |
| 1 + Session | 0.15 | 0.28 | 0.01 |
| 2 + Sessions | 0.14 | 0.30 | 0.01 |
| 3 + Sessions | 0.13 | 0.37 | 0.01 |
| 4 + Sessions | 0.19 | 0.17 | 0.02 |
| 5 + Sessions | 0.18 | 0.19 | 0.02 |
| 6 + Sessions | 0.15 | 0.32 | 0.01 |

**Supplementary Material 3.** **PP estimation with inverse probability weighting for censoring.** For this analysis we estimate inverse probability weighting for completer participants (which are defined as attending 4 sessions or more) for both in-person and remote modalities. First, we ran logistic regression models predicting completion (4+ sessions) vs no completions (less than 4 sessions) with demographic variables, mental health outcomes, and subscales of the brief COPE. We selected the significant and “threshold” significant predictors (p <0.1) and estimated inverse probability weights for participants using the “ipwpoint” function from the “ipw” package in R including said variables.

- Variables included for the in-person modality: Civil status, ethnicity, nationality, labor status, gender, age, active coping, humor, self-blame, and venting.
- Variables included for the remote modality: Civil status, nationality, labor status, gender, age, WHODAS, information support, planning, humor, and emotional support.

Since session attendance is only a criterion valid for participants in the experimental condition, we do not have a way to build a “completion” censoring variable for participants in the waitlist group. Therefore, we opt to use a “non-informative” inverse probability weight of 1 for all waitlist participants. Results for a multilevel regression with individual as random effect, fixed effects for treatment, time, and the interaction of treatment and time with inverse probability weight at the individual level are shown here:

|  | **PP with Inverse Probability Weighting** | | | |
| --- | --- | --- | --- | --- |
| *Model* | *Treatment Effect* | *p* | ɳ^2^_p_ | *ICC* |
| *In Person modality* |  |  |  |  |
| Depression | **-0.35** | **0.0001** | **0.12** | 0.62 |
| Anxiety | **-0.35** | **0.0001** | **0.11** | 0.65 |
| PTSD | **-0.32** | **0.0015** | **0.08** | 0.55 |
| Generalized distress | -0.27 | 0.0809 | 0.02 | 0.41 |
| Functional impairment | -0.05 | 0.4488 | 0.00 | 0.52 |
| Community resilience | -0.01 | 0.9355 | 0.00 | 0.53 |
| *Remote modality* |  |  |  |  |
| Depression | 0.10 | 0.54 | 0.00 | 0.42 |
| Anxiety | 0.03 | 0.85 | 0.00 | 0.54 |
| PTSD | 0.22 | 0.12 | 0.03 | 0.62 |
| Generalized distress | -0.06 | 0.77 | 0.00 | 0.30 |
| Functional impairment | -0.04 | 0.75 | 0.00 | 0.64 |
| Community resilience | 0.30 | 0.08 | 0.04 | 0.21 |

Results shown here seem even less conservative than those encountered by the non-weighted inverse probability estimation. Therefore, we choose not to report them in the manuscript since we have concerns about the adequacy of inverse probability weighting when the control group has no censoring variable to estimate these weights on.
